# Relationship Between Display Pixel Structure and Gloss Perception

**DOI:** 10.3390/jimaging12020071

**Published:** 2026-02-09

**Authors:** Kosei Aketagawa, Midori Tanaka, Takahiko Horiuchi

**Affiliations:** 1 Graduate School of Science and Engineering, Chiba University, Yayoi-cho 1-33, Inage-ku, Chiba 263-8522, Japan; 2 Graduate School of Informatics, Chiba University, Yayoi-cho 1-33, Inage-ku, Chiba 263-8522, Japan; horiuchi@faculty.chiba-u.jp

**Keywords:** subpixel array, pixel–aperture ratio, shitsukan, appearance, glossiness, display technology, gloss perception, high-definition

## Abstract

The demand for accurate representation of gloss perception, which significantly contributes to the impression and evaluation of objects, is increasing owing to recent advancements in display technology enabling high-definition visual reproduction. This study experimentally analyzes the influence of display pixel structure on gloss perception. In a visual evaluation experiment using natural images, gloss perception was assessed across six types of stimuli: three subpixel arrays (RGB, RGBW, and PenTile RGBG) combined with two pixel–aperture ratios (100% and 50%). The experimental results statistically confirmed that regardless of pixel–aperture ratio, the RGB subpixel array was perceived as exhibiting the strongest gloss. Furthermore, cluster analysis of observers revealed individual differences in the effect of pixel structure on gloss perception. Additionally, gloss classification and image feature analysis suggested that the magnitude of pixel structure influence varies depending on the frequency components contained in the images. Moreover, analysis using a generalized linear mixed model supported the superiority of the RGB subpixel array even when accounting for variability across observers and natural images.

## 1. Introduction

Human perception of an object’s appearance and quality relies on visual information derived from the way surface optical properties interact with surrounding illumination [1]. A central concept in this context is the Japanese term shitsukan, which has no direct one-to-one equivalent in English [2]. The word combines shitsu (quality) and kan (sensation) and is used to describe the overall impressions of an object that arise from its material and quality. From visual appearance alone, humans can judge an object’s condition (e.g., dry or wet), infer what it is made of (e.g., wood or glass), and identify surface qualities such as glossy, rough, or transparent. In addition to such physical material properties estimated through visual processing, shitsukan also encompasses the subjective feelings evoked by objects, including comfort or discomfort, and evaluations of biological, emotional, aesthetic, or social value. These higher-level psychological responses are strongly modulated by the characteristics, preferences, and past experiences of the observer. For example, just as color temperature psychologically affects the perceived warmth of a space beyond its physical lighting characteristics, shitsukan perception is grounded in physical visual cues yet shaped by psychological interpretation. Recent studies in the fields of information engineering, psychophysics, and neuroscience have advanced our understanding of shitsukan [3], highlighting the growing importance of its accurate reproduction and communication.

Among the visual shitsukan attributes, gloss perception is an impression based on surface reflection characteristics and is composed of multiple visual cues, including the ratio of specular to diffuse reflection, the distribution of reflection highlights, and spatial frequency characteristics. Motoyoshi et al. [4] demonstrated the relationship between image statistics and gloss perception, revealing that the intensity distribution of reflected light directly influences gloss perception. Fleming [5] further noted that material perception, including gloss, arises from the visual system’s construction of statistical appearance models based on image features rather than direct estimation of physical properties such as reflectance or bidirectional reflectance distribution function parameters. These studies indicate that gloss perception is a complex phenomenon that depends not only on physical properties but also on observers’ experiences and cognitive factors.

Furthermore, advances in display technology have increased the demand for the accurate reproduction of shitsukan in display-presented images. Although conventional displays define image resolution using pixel count, recent findings indicate that perceptual resolution can vary even among displays with identical nominal resolutions, depending on differences in pixel structure. Our prior investigations demonstrated that variations in pixel–aperture ratio (100% vs. 3%) affect perceptual spatial resolution [6], establishing that physical pixel configuration influences visual impressions. Subsequently, we conducted experimental studies to examine the effect of different subpixel arrays (RGB, RGBW, and PenTile RGBG) on the perception of roughness, gloss, and transparency, confirming that subpixel array variations affect shitsukan perception [7,8].

However, the pixel–aperture ratio conditions employed in these previous studies were extreme (100% or 3%) and failed to adequately represent practical display configurations. In actual display systems, pixel structures with an aperture ratio of 100% rarely exist, and the combined effects of subpixel arrays and pixel–aperture ratios, two distinct structural elements, on shitsukan perception remain insufficiently explored. Recently, the OLED display market has expanded substantially, with mainstream OLED panels featuring distinctive pixel architectures characterized by moderate aperture ratios [9]. Consequently, elucidating the influence of moderate pixel–aperture ratio conditions, which better reflect realistic display structures, on gloss perception, remains a crucial challenge in the field of display engineering.

To address these challenges, this study aimed to experimentally examine the impact of pixel structure on gloss perception using stimuli featuring three subpixel array types (RGB, RGBW, and PenTile RGBG) combined with two pixel–aperture ratios (100% and 50%). We analyzed the effect of variations in subpixel arrays and pixel–aperture ratios on gloss perception through subjective evaluations conducted by observers. Based on the experimental results, we performed statistical hypothesis testing, effect size analysis, observer clustering, and image feature analysis to systematically investigate the relationship between pixel structure and gloss perception. Additionally, unlike previous studies, we conducted a generalized linear mixed model (GLMM) analysis to account for variability across observers and images, enabling a more robust estimation of the fixed effects of pixel structure on gloss perception.

The remainder of this paper is organized as follows. Section 2 describes the experimental methods, including stimulus generation and the experimental procedure, and reports the modulation transfer function (MTF) calculated for the generated stimuli. Section 3 presents the results, including analysis of the mean response rates across all observers, observer cluster analysis, image feature analysis, and GLMM analysis. Section 4 provides the conclusions and discusses implications for display design, limitations, and future work, thereby concluding the paper.

## 2. Experiment

To analyze the effect of pixel structure differences on gloss perception in displayed natural images, stimuli with six types of pixel structures were created using five natural images and gloss perception was visually evaluated by observers. In addition, to characterize the physical properties of the generated stimuli, we present a modulation transfer function (MTF) analysis as a quantitative measure of their spatial-frequency characteristics.

### 2.1. Experimental Stimuli

**Figure 1:**
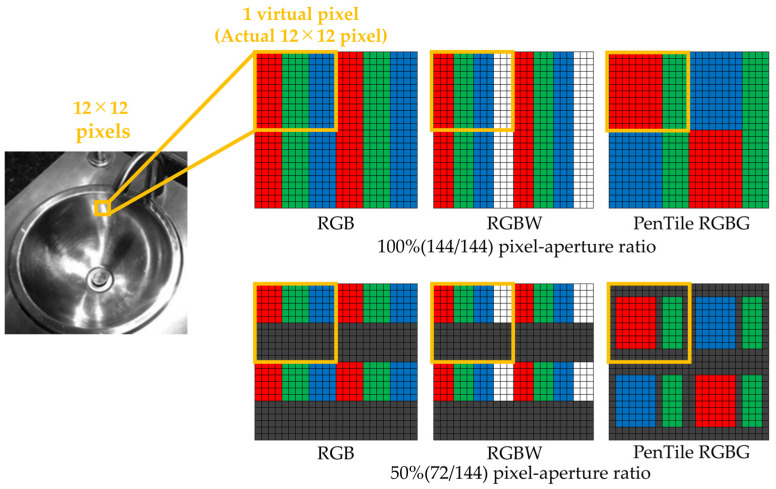
Construction of the virtual pixel structure for experimental stimuli.

The experimental stimuli were generated using a simulation method from previous studies [6,7,8] to create stimuli with different pixel–aperture ratios. First, the generation method is described. The OLED display used in this experiment (FORIS NOVA, EIZO Corp., Ishikawa, Japan) features an RGB subpixel array. Additionally, preparation of multiple displays that differ only in pixel structure conditions is challenging. Therefore, six types of pixel structures were realized via simulation by treating 12 × 12 pixels of the actual display as a single virtual pixel. Figure 1 shows the configuration of the six pixel structures used in this experiment. Hereafter, each pixel structure is referred to by its abbreviation (RGB subpixel array with 100% pixel–aperture ratio → RGB100%, PenTile RGBG → PenTile). To standardize the virtual pixel width of each pixel structure, the virtual subpixel widths were set as follows: RGB100% at 4 × 12 pixels, RGBW100% at 3 × 12 pixels, PenTile100% with R and B at 8 × 12 pixels and G at 8 × 12 pixels, RGB50% at 4 × 6 pixels, RGBW50% at 3 × 6 pixels, and PenTile50% with R and B at 6 × 8 pixels and G at 3 × 8 pixels. Notably, PenTile reproduces RGB using two virtual pixels. Additionally, a spectroradiometer (CS-2000, Konica Minolta, Inc., Tokyo, Japan) was used to adjust the pixel values such that the average luminance of the experimental stimuli remained uniform across the six stimuli with different pixel structures. This ensured that the differences in brightness between the stimuli did not affect the evaluation. The maximum luminance of the experimental stimuli for each pixel–aperture ratio was set such that white consistently measured at 52.2 cd/m^2^. The 12 × 12 pixels of the experimental stimuli created using this procedure were treated as single virtual pixels. When evaluating the experimental stimuli with six different pixel structures, one virtual pixel was observed from a viewing distance 12 times that of a single real pixel. Section 2.3 presents the modulation transfer function (MTF) analysis as a measure of the physical characterization of the generated stimuli.

**Figure 2:**
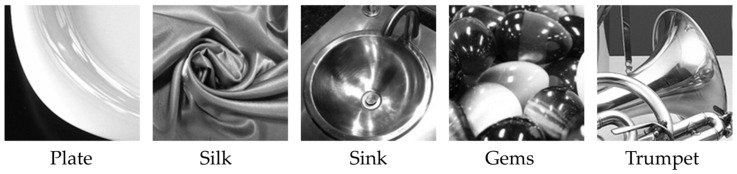
Image content used as experimental stimuli.

Next, we describe the natural images used in the experiment. Five natural images were obtained from standard image collections (SCID and SHIPP), the Flickr Material Database [10], and the Describable Textures Dataset [11]. In this study, images captured from real-world objects are referred to as “natural images,” to distinguish them from computer-generated images and artificial pattern stimuli such as gratings or random dots. Each image was cropped to dimensions of 150 × 150 pixels to preserve adequate visual content—specifically, sufficient gloss information corresponding to Hunter’s six-category gloss taxonomy [12]—necessary for shitsukan assessment and then converted to grayscale using the National Television System Committee formula to remove color-related effects on gloss perception. Our previous study demonstrated that color modulates shitsukan impressions, with findings indicating that attributes such as paper’s “glossiness” and rubber’s “aesthetic appeal” are perceived differently in color versus grayscale representations [13]. These observations indicate that color exerts a substantial influence on shitsukan evaluation; consequently, we adopted grayscale imagery for the present investigation. Experimental stimuli were generated by scaling each original pixel of the cropped images (150 × 150 pixels) by 12 × 12, yielding final stimuli measuring 1800 × 1800 display pixels (equivalent to 150 × 150 virtual pixels). Figure 2 presents five representative natural images used to evaluate the gloss perception. The stimulus set incorporated diverse gloss types corresponding to Hunter’s six-category gloss taxonomy [12].

### 2.2. Experimental Procedure

In this experiment, a side-by-side protocol, commonly used in visual evaluation studies [14], was adopted. Stimulus pairs simulating different pixel structures were presented on the left and right sides of a black background on the display. Observers were asked to evaluate the stimulus that exhibited stronger gloss perception using the two-alternative forced choice (2AFC) method. Observers were instructed to select “the one that appears glossier.” This instruction was thoroughly explained both verbally and in writing before the experiment began, and we confirmed that all participants understood the task clearly. In this briefing, “gloss” was explained as a visual impression arising from surface reflectance characteristics, referring to Hunter’s six-category gloss taxonomy [12]. Prior to evaluation, observers were also informed of the object names depicted in each stimulus image, and we confirmed that they had a sufficient understanding of the visual content. Twelve stimulus pairs with different pixel structure combinations were created for each type of natural image to examine the effects of pixel structure differences on gloss perception. Pairs consisting of 100% pixel–aperture ratio conditions were excluded because they overlapped with our previous study [7]. Table 1 presents specific stimulus pair combinations. Hereafter, the stimulus pairs mentioned in the text are denoted by their Pair IDs listed in Table 1. For example, P01 represents a stimulus pair comparing RGB100% vs. RGB50%.

In this experiment, fifteen observers (14 males and 1 female; mean age 23.4 ± 2.3 years) participated. All observers were undergraduate or graduate students. The observers first completed a standard Landolt C visual acuity test and the Ishihara color vision test, confirming binocular visual acuity equivalent to 20/20 and normal color vision, followed by a 5 min dark adaptation period. After the adaptation period, the stimulus pairs were presented on the left and right sides of a display with a black background in a dark room. For each stimulus pair, the observers selected the stimulus that appeared glossier. Following each response, a black background was displayed for 1 s to eliminate any influence of the previously evaluated stimulus. Subsequently, the next stimulus pair was presented and the evaluation process was repeated. The viewing distance was set to 30 cpd (5.14 m), which is equivalent to the standard viewing distance recommended by ITU-R [15], the Radiocommunication Sector of the International Telecommunication Union. The pixel pitch of the OLED display used in this experiment was 0.0001245 m. A spatial frequency of 30 cycles per degree (cpd) corresponds to the viewing condition in which two adjacent pixels subtend an angle of 1 arc-min at the viewer’s eye. Therefore, the viewing distance D was calculated using the following equation:

(1) D=12×0.0001245×30×sin89.5°/sin0.5°≅5.14 [m]

Here, because a 12×12 pixel structure was treated as one virtual pixel in this experiment, the pixel pitch was multiplied by 12. Each pair was evaluated 16 times to ensure reliability. To counter the effects of display luminance nonuniformity, each stimulus pair was presented equally often in the two left–right orders. For example, for the RGB100% vs. RGB50% pair, “left: RGB100%, right: RGB50%” was presented 8 times and the reversed order “left: RGB50%, right: RGB100%” was presented 8 times (16 trials in total). This design cancels out any position-related luminance advantage in the aggregated choice rate (e.g., even if an observer tends to choose the right side due to luminance nonuniformity, the overall choice rate converges to 50:50). Throughout the experiment, each observer completed 960 evaluations (12 pairs × 16 evaluations per pair × 5 stimulus types). The order of the stimulus presentation was randomized to minimize bias.

### 2.3. Modulation Transfer Function Calculation

The MTF represents the magnitude of response to sinusoidal waves of different spatial frequencies and provides objective and quantitative spatial frequency characteristics of displays. In a previous study [8], the MTF was employed to analyze and discuss perceptual resolution. In this study, we calculated the MTF [16] to also account for differences in physical conditions, such as the pixel–aperture ratio, and discussed the results alongside the evaluation findings. Using MTF is expected to be effective because it enables a quantitative analysis of the influence of spatial frequency characteristics owing to pixel structure on gloss perception. Shishikui et al. [17] reported that image resolution affects both perceptual resolution and lower-order visual impressions such as color vividness, gloss, and depth perception, with these impressions strengthening as image resolution increases. Furthermore, our previous study [8] revealed that a higher MTF corresponds to a higher perceptual resolution. These findings indicate that gloss perception also strengthens as the MTF increases. For example, the line spread function $LSF\left(x\right)$ of the luminance profile in the vertical projection for a 100% pixel–aperture ratio with an RGB subpixel array is as follows:

(2) $$LSF\left(x\right)=2\left(\frac{L_{R}}{L_{RGB}}rect\left(\frac{x+\frac{5}{6}}{\frac{1}{3}}\right)+\frac{L_{G}}{L_{RGB}}rect\left(\frac{x+\frac{3}{6}}{\frac{1}{3}}\right)+\frac{L_{B}}{L_{RGB}}rect\left(\frac{x+\frac{1}{6}}{\frac{1}{3}}\right)\right),$$

Here, rect represents a rectangular function. $L_{R}$, $L_{G}$, and $L_{B}$ denote the luminance of the red, green, and blue subpixels, respectively, and $L_{RGB}$ is defined as $L_{RGB}\;$= $L_{R}+L_{G}+L_{B}$. The $MTF\left(\xi \right)$ calculated by performing a Fourier transform on the obtained $LSF\left(x\right)$ and normalizing it to equal 1 at ξ=0 is as follows:

(3) $$MTF\left(\xi \right)=\left|sinc\left(\frac{1}{3}\xi \right)\left(\frac{L_{R}}{L_{RGB}}e^{j\frac{5}{3}\pi \xi }+\frac{L_{G}}{L_{RGB}}e^{j\pi \xi }+\frac{L_{B}}{L_{RGB}}e^{j\frac{1}{3}\pi \xi }\right)\right|,$$

**Figure 3:**
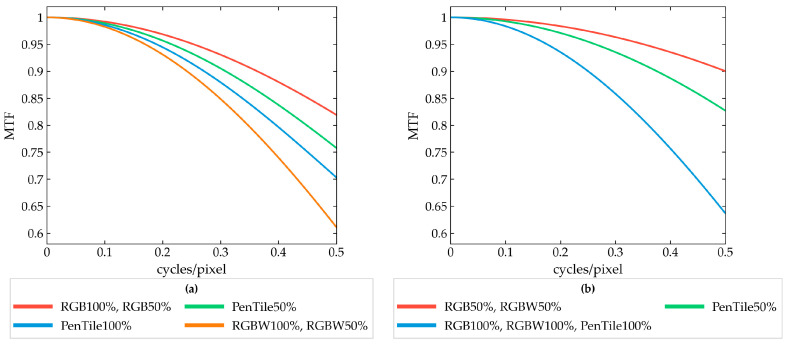
Calculated MTF for each pixel structure: (**a**) vertical projection and (**b**) horizontal projection.

Figure 3 shows the vertical and horizontal projection MTFs for each pixel structure, calculated using the same procedure. Based on the MTF values, the superiority relationships among the pixel structures were as follows: for vertical projection, RGB100% = RGB50% > PenTile50% > PenTile100% > RGBW100% = RGBW50%; for horizontal projection, RGB50% = RGBW50% > PenTile50% > RGB100% = RGBW100% = PenTile100%.

## 3. Results and Discussion

This section details the results obtained from the visual evaluation experiment and analyzes the effects of each pixel structure on gloss perception along with the result of MTF calculation. Additionally, we discuss the influence of individual observer response tendencies and image characteristics on the results. Comparisons were made with our previous study [6], which investigated the effects of three subpixel array types (RGB, RGBW, and PenTile RGBG) on gloss perception. Finally, we employed a GLMM to treat the variability across observers and natural images as random effects, thereby estimating the fixed effects of pixel structure on gloss perception, which is common under different conditions.

### 3.1. Response Rate and Significant Differences

**Figure 4:**
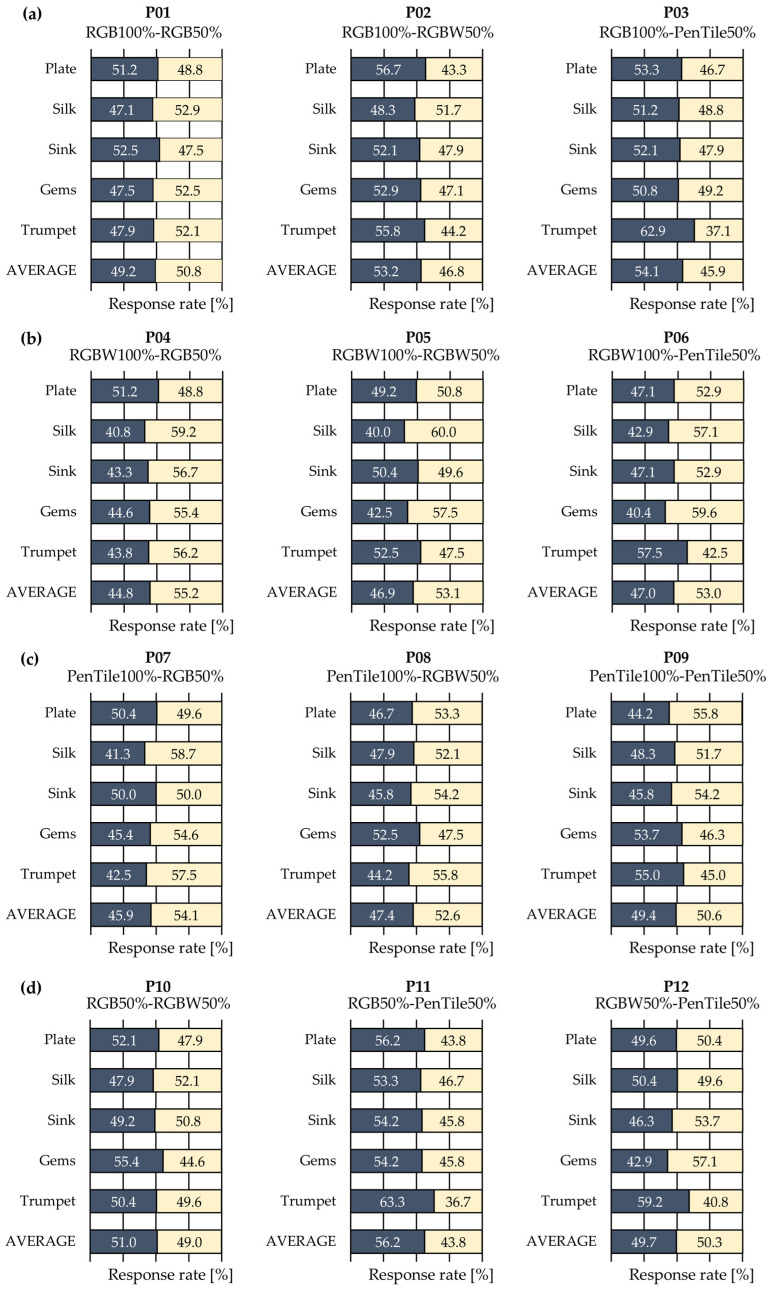
Average response rates of 15 observers. The figure is divided into (**a**–**d**) for stimulus pairs P01–P12: (**a**) RGB100% vs. 50% conditions (P01–P03), (**b**) RGBW100% vs. 50% conditions (P04–P06), (**c**) PenTile100% vs. 50% conditions (P07–P09), and (**d**) 50% vs. 50% conditions (P10–P12).

Figure 4 shows the average response rates of the 15 observers who reported a stronger gloss perception. The average response rate was calculated as follows: for example, in P01 (RGB100–RGB50%), if one observer selected RGB100% six times and RGB50% ten times, the response rate for RGB100% was 6/16 (37.5%) and for RGB50% it was 10/16 (62.5%). The same calculation was performed for all of the 15 observers and the average response rate for each natural image was calculated based on their response rates. Additionally, statistical *p*-values, standard deviations, effect sizes, and effect size indices were calculated and analyzed, as shown in Table 2, to determine whether significant differences existed in gloss perception owing to pixel structure variations. In this study, a two-sample *t*-test was employed as the analytical method, and Cohen’s *d* was calculated as a measure of effect size. For the *t*-test and effect size calculation, the response rates of the 15 observers for each natural image were used (*n* = 15 observers × 5 natural image types = 75). In the table, statistical significance is indicated using asterisks (*: *p* < 0.05, **: *p* < 0.01, ***: *p* < 0.001). Effect size indices were selected based on the closest match according to Sawilowsky and Cohen [18,19]: Very Small (*d* < 0.105), Small (0.105 ≤ *d* < 0.35), Medium (0.35 ≤ *d* < 0.65), Large (0.65 ≤ *d* < 1.00), Very Large (1.00 ≤ *d* < 1.60), and Huge (*d* ≥ 1.60). For instance, when the effect size was large, it was classified in stages such as “Large,” “Very Large,” or “Huge,” indicating a perceptible difference in gloss perception between stimulus pairs. This method enabled the evaluation of the effect magnitude independent of the sample size.

Based on the results in Table 2, focusing on the *p*-values that indicate whether significant differences existed between the average response rates, statistical significance was confirmed for the six pairs. Additionally, examining effect sizes independent of sample size, pairs P04 and P11 were classified as “Large;” however, “Very Large” or “Huge” classifications did not appear. By examining the superiority relationships among pixel structures based on the average response rates of the six pairs showing statistical significance, Figure 4 shows that the RGB subpixel array was perceived as exhibiting stronger gloss than PenTile and RGBW regardless of the pixel–aperture ratio. Except for P05, all pairs showing statistical significance included either RGB100% or RGB50%. In P05, which compared the RGBW conditions, RGBW50% with a lower pixel–aperture ratio was superior.

Consequently, among pairs showing statistical significance, the order of perceived gloss strength was RGB > PenTile ≈ RGBW for subpixel arrays and 50% > 100% for aperture ratios. In particular, RGB50%, which exhibited the highest MTF in both vertical and horizontal directions, was perceived to have the strongest gloss, which was generally consistent with the MTF predictions. In our previous study [7], which analyzed the effects of subpixel arrays on gloss perception under 100% pixel–aperture ratio conditions, the superiority relationship for gloss perception was RGB100% > PenTile100% > RGBW100%, which is consistent with the vertical projection MTF superiority relationship (the horizontal projection MTF at 100% pixel–aperture ratio is equivalent regardless of the subpixel array). However, in cases where the superiority relationships differed between the vertical and horizontal projection MTFs, such as RGB100% vs. PenTile50%, RGB100% with a higher vertical projection MTF was superior. Conversely, for pairs differing only in aperture ratio, such as P01, P05, and P09, significant differences appeared only in P05, whereas P01 and P09 showed small effect sizes. This observation highlights the fact that vertical and horizontal MTFs alone cannot fully explain gloss perception.

Next, we briefly introduce the results obtained by the individual observers. For example, Observer 8 showed only one pair with “Huge” effect size or significant *p*-value among all 12 pairs, whereas Observer 13 showed five such pairs, confirming that the effects of pixel structure differences vary substantially among observers. Individual differences also existed in the pairs, showing significant and superior relationships among pixel structures. These results confirmed that the perception of gloss is influenced by the differences in pixel structure. However, substantial individual variability was observed in the observer responses. Such variations in individual evaluations may stem from observers’ subjective assessments. Therefore, when evaluating the effects of pixel–aperture ratio differences on shitsukan, the differences in perceptual characteristics and evaluation criteria among the observers must be considered. Additionally, individual differences in perceived shitsukan may be influenced by each observer’s learning experiences and response tendencies to stimuli. To further investigate these individual differences in response tendencies, a cluster analysis was conducted in the following section.

### 3.2. Cluster Analysis—Observer Classification

**Figure 5:**
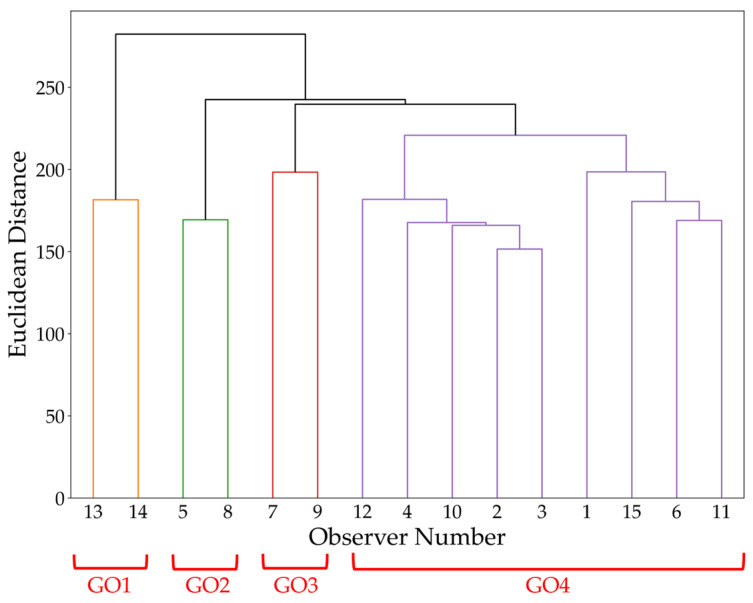
Dendrogram of observer cluster classification. Cluster labels (GO1–GO4) are shown in red brackets.

**Figure 6:**
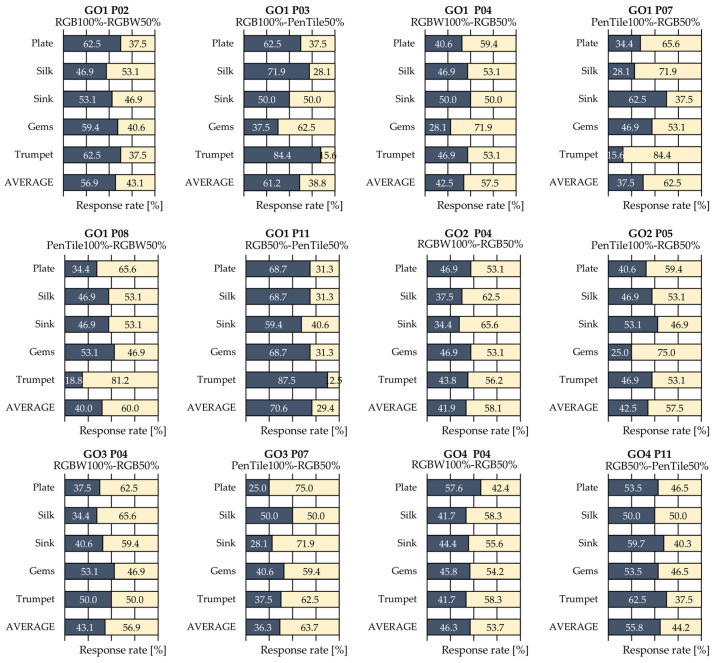
Average response rates for each cluster (only pairs showing significant *p*-values or effect sizes of “Very Large” or greater).

A cluster analysis was conducted to classify the observers and account for individual response tendencies, based on the results of the visual evaluation experiment. This analysis aimed to group observers based on their response data and clarify the differences in perception tendencies, as individual observers may exhibit varying response patterns. Hierarchical clustering (Ward’s method) was used for the analysis. Clustering utilized 60-dimensional response rate data per observer (5 different image contents × 12 pairs). A dendrogram is shown in Figure 5, in which the observers were divided into four clusters (designated as GO1, GO2, GO3, and GO4 from left to right). Additionally, Table 3 and Figure 6 show the average response rates for pairs exhibiting significant *p*-values or effect sizes of “Very Large” or greater in each cluster, along with the significance indicated by effect sizes. Owing to space limitations, only pairs showing significant differences or effect sizes of “Very Large” or greater are presented.

As seen in Table 3, in GO1–GO3, wherein each cluster contains two observers with equal sample sizes, statistical significance appeared in P11 for GO1 and P07 for GO3, both confirming “Huge” effect sizes. Other pairs showed “Very Large” effect sizes without statistical significance and GO1 exhibited “Very Large” effect sizes in five pairs, a greater number than GO2 and GO3. This reveals the differences in the effects on gloss perception among individual observers. In GO4, which contains nine observers, significant *p*-values were confirmed for both P04 and P11, which were classified as “Large”—the largest effect size in Section 3.1. Notably, P04, which showed the greatest MTF difference in both the vertical and horizontal directions, exhibited a “Very Large” effect size and a significant *p*-value across all clusters. Furthermore, the superiority relationships among the pixel structures across all pairs in Figure 6 were confirmed to be consistent with the average results for all observers described in Section 3.1.

These results suggest that individual differences exist in the effects of pixel structure variations on gloss perception. Specifically, although observers as a group generally exhibited consistent trends, the magnitude of the effects and degree of response for individual stimulus pairs varied among individuals, suggesting that individual observers may differ in their sensitivity to gloss judgments and the factors they focus on.

### 3.3. Image Classification and Image Features

In addition to the differences in pixel structure, the image features of the five natural images were calculated to investigate the image characteristics, which influenced the observer response tendencies. The image features used included contrast (an index of local luminance variation) and energy (an index of texture repetition) from the gray-level co-occurrence matrix (GLCM) [20], which is effective for texture analysis of objects in images, as well as kurtosis and skewness calculated from the image luminance histogram. Kurtosis represents the sharpness or flatness of the peaks, whereas skewness indicates the asymmetry of the distribution; these are widely used to effectively capture texture characteristics [21]. Additionally, to examine the influence of the frequency components contained in the images on the evaluation, the mean frequency, which was used in our previous study [7], was also calculated. The mean frequency is the average of the frequencies weighted by the amplitude of each frequency component. Higher values indicate that the image contains more high-frequency components. Furthermore, root mean square (RMS) contrast, which has been reported to correlate with gloss perception in natural images, was calculated. The RMS contrast is defined as the standard deviation of pixel values, and Wiebel et al. [22] demonstrated in experiments using natural images that contrast strongly correlates with gloss perception.

**Figure 7:**
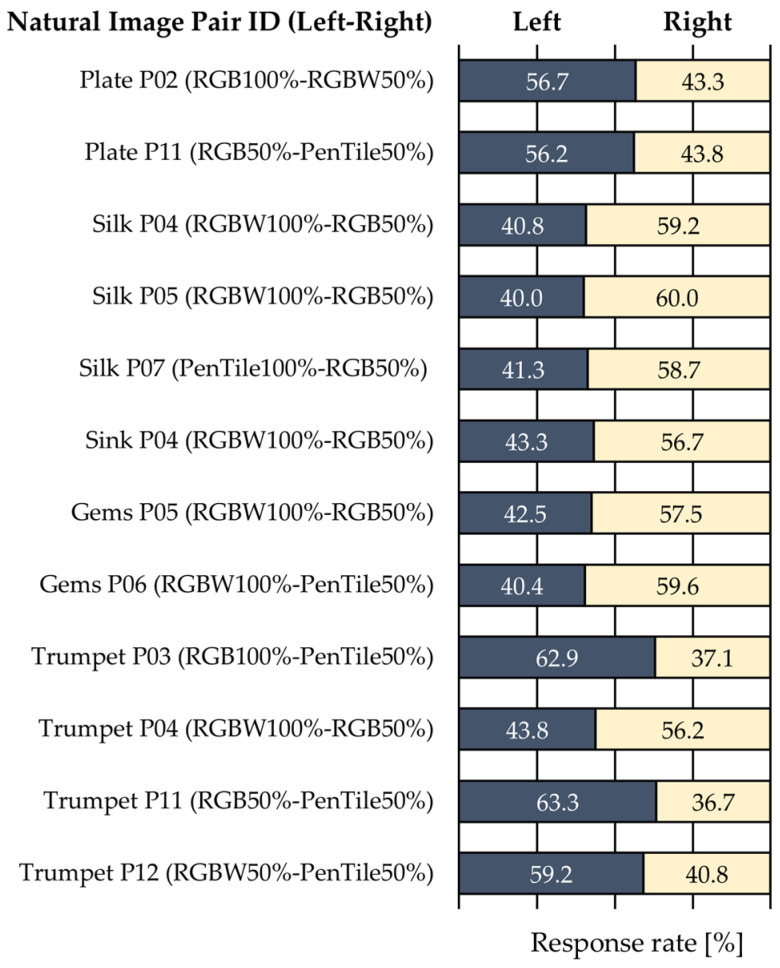
Average response rates for each natural image (only pairs showing significant *p*-values or effect sizes of “Very Large” or greater).

Table 4 presents the calculated image features for each natural image. Additionally, Figure 7 and Table 5 show the average response rates for pairs exhibiting significant *p*-values or effect sizes of “Very Large” or greater for each natural image, along with the significance indicated by effect sizes. For each natural image, the gloss was classified as follows, based on the features and Hunter’s six gloss categories [12]:

#### 3.3.1. Plate

With an overwhelmingly high GLCM Energy and the lowest GLCM contrast, this image exhibited an overall uniform texture and was monotonous. The normalized mean frequency was 0.751, indicating relatively few high-frequency components. The RMS contrast was the highest, indicating a large luminance variation across the image. The lowest skewness suggested that the image was relatively bright overall, and the gloss was classified as a specular gloss. Two stimuli pairs showed significant *p*-values or effect sizes of “Very Large” or greater.

#### 3.3.2. Silk

With the lowest GLCM energy and relatively low GLCM contrast, this image was not uniform but exhibited low luminance differences between pixels. The RMS contrast was lowest at 0.551, indicating a small luminance variation across the image. A relatively high kurtosis suggests a small pixel value variation, and the gloss was classified as sheen. However, mean frequency was 0.904, the second highest, indicating many high-frequency components, and three stimuli pairs showed significant *p*-values or effect sizes of “Very Large” or greater.

#### 3.3.3. Sink

With the highest skewness and kurtosis, moderate GLCM contrast, and relatively low GLCM energy (second from the bottom), this image exhibited bright gloss spread across the surface, although the overall image was relatively dark. Its appearance was dominated by the contrast between strong specular reflections on the metal and dark areas, classifying the gloss as contrast gloss. Mean frequency was moderate (third), and one stimulus pair showed a significant *p*-value or effect size of “Very Large” or greater.

#### 3.3.4. Gems

Although the GLCM contrast was relatively high, the mean frequency was the lowest, indicating luminance differences in fine highlighted areas but a smooth surface with the most low-frequency components. The gloss was classified as specular gloss, and two stimulus pairs showed significant *p*-values or effect sizes of “Very Large” or greater.

#### 3.3.5. Trumpet

With an overwhelmingly high GLCM contrast and the highest mean frequency, this image had many high-frequency components with numerous edges and complex textures. Gloss was classified as a distinctness-of-image gloss, characterized by clear reflection images, based on appearance. Four stimulus pairs showed significant *p*-values or effect sizes of “Very Large” or greater.

Synthesis of the analysis results of individual images revealed that the mean frequency was the index that best explained the tendency of the significant differences observed in this study’s evaluation. Trumpet, with the highest mean frequency, showed the most significant differences with two “Huge” pairs and two “Very Large” pairs. For Silk, the mean frequency rank (second) matched the number of significant differences rank (second). These results suggested that images containing more high-frequency components were more likely to exhibit greater effects of pixel structure differences on gloss perception. Conversely, the sink and gems showed reversed rankings between the mean frequency and the number of significant differences. However, the sink, with the third-highest mean frequency, although having the lowest number of significant differences, achieved a “Very Large” effect size for the RGB50–RGBW100% stimulus pair, which had the largest vertical and horizontal MTF difference. By contrast, the plate and gems, with mean frequencies ranked fourth and fifth, respectively, showed effect sizes of only “Small” and “Large” for the same RGB50–RGBW100% stimulus pair, with relatively smaller effects owing to MTF differences. Notably, gems with the lowest mean frequency showed no significant differences in stimulus pairs, including the RGB subpixel array, despite the overall average, indicating that the RGB subpixel array was perceived as having the strongest gloss.

These results suggested that differences in the frequency components contained in the images may be related to the magnitude of the effects on gloss perception owing to the MTF differences between pixel structures. Specifically, images with more high-frequency components were more likely to show larger effects from MTF differences, whereas images with relatively more low-frequency components exhibited smaller effects. The results of this study suggested that the interaction between the mean frequency and MTF differences may contribute to the influence of pixel structure on gloss perception. Further verification, targeting diverse image conditions and stimulus structures, is expected to provide a clearer understanding of this relationship.

### 3.4. Estimation of Overall Trends Using Generalized Linear Mixed Model

In Section 3.1, Section 3.2 and Section 3.3, *t*-tests for individual stimulus pairs, observer clustering, and image feature analysis were conducted separately. However, these analyses make it difficult to handle individual differences among observers and the variability across natural images within a unified framework. Therefore, in this section, we employed a GLMM to incorporate the variability across observers and natural images as random effects and estimated the overall trends in gloss perception across pixel structures. In psychophysical experiments, the GLMM has been used to integrate data from multiple observers [23]. For this analysis, we adopted a binomial GLMM based on the Bradley–Terry model [24], which is widely used to analyze paired comparison data. In this study, we assumed a binomial distribution and logit link function for responses, with pixel structure conditions as fixed effects and observers (*N_obs_* = 15) and natural images (*N_img_* = 5) as random effects (random intercepts). The reference stimulus was set to RGBW100%, which was suggested to have the weakest gloss perception based on the response rates and *t*-test results in Section 3.1. The analysis was performed using R (version 4.5.2) and the lme4 package (version 1.1-37) [25].

**Figure 8:**
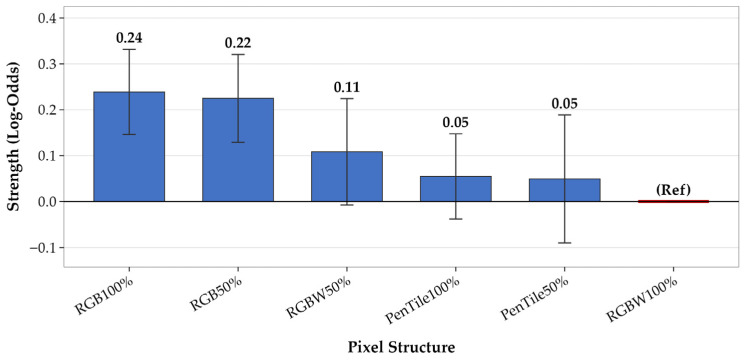
Estimated glossiness strength (β) and 95% confidence intervals for each pixel structure.

Table 6 shows the fixed effect estimation results, and Figure 8 shows the estimated strength β and 95% confidence intervals. The estimated value β in Table 6 represents the gloss intensity of each stimulus in log-odds, as a relative value to the reference stimulus RGBW100% (β = 0). Larger positive values indicate a stronger perceived gloss than that of the reference. Standard error (Std. Err) represents the uncertainty of the estimate, and *p*-values were calculated based on the Wald test. In Figure 8, the error bars represent 95% confidence intervals; when this interval does not cross zero (the value of the reference stimulus), a statistically significant difference exists from the reference stimulus. As shown in Table 6 and Figure 8, RGB100% and RGB50% exhibited significantly higher gloss intensities than the reference stimulus RGBW100%. By contrast, the differences from the reference stimuli for RGBW50%, PenTile100%, and PenTile50% were not significant.

**Figure 9:**
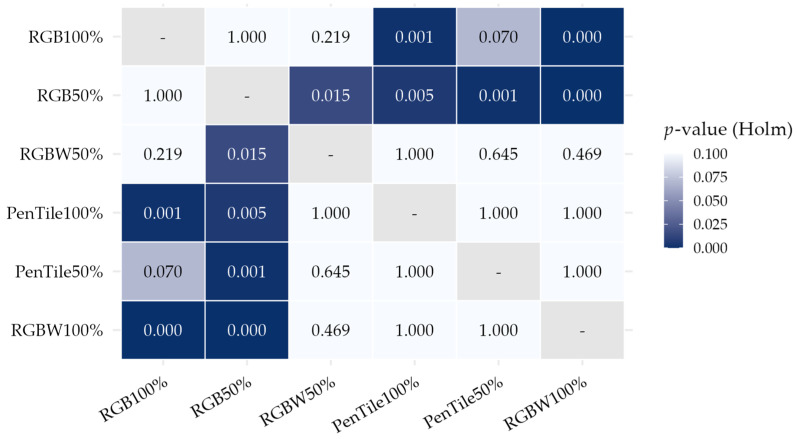
Heatmap of Holm-corrected *p*-values for pairwise comparisons between pixel structure conditions.

Next, to evaluate the relationships between pairs, we applied Holm’s method for multiple comparison correction to the original *p*-values based on Wald tests for all stimulus pairs. The corrected *p*-values are shown as a heatmap in Figure 9. All values shown in Figure 9 are Holm-corrected *p*-values, which serve as indices for determining the significance of each pair while accounting for multiple comparisons. Although Figure 8 is limited to comparisons with the reference stimulus RGBW100%, Figure 9 shows all 15 pairwise comparisons. As shown in Figure 9, no significant difference was observed between RGB100% and RGB50% (*p* = 1.000), indicating similar gloss intensities. However, RGB50% exhibited significant differences with four conditions: RGBW100%, PenTile100%, RGBW50%, and PenTile50%. RGB100% exhibited significant differences with only two conditions: RGBW100% and PenTile100%. Therefore, although RGB100% and RGB50% showed similar gloss intensities in direct comparison, RGB50% was more distinct from the perspective of statistical separation from other pixel structures.

Examining the variance components of random effects, the variance across natural images (σ^2^ = 0.006) was somewhat larger than the variance across observers (σ^2^ = 0.002), suggesting that differences in natural image content exert a relatively larger influence on gloss evaluation than that by observers. Additionally, the dispersion ratio (1.077) was close to one, indicating no major issues with the model fit.

The results of this GLMM analysis were consistent with the *t*-test analysis results presented in Section 3.1, supporting the overall trend that the RGB subpixel array was perceived as having the strongest gloss, regardless of the pixel–aperture ratio, and that RGB50% was perceived as having the strongest gloss, even when accounting for variability across observers and natural images.

## 4. Conclusions

This study experimentally analyzed the influence of display pixel structure on gloss perception using six types of stimuli combining three subpixel arrays (RGB, RGBW, and PenTile) with two pixel–aperture ratios (100% and 50%). Unlike previous studies that examined subpixel arrays or aperture ratios separately under extreme conditions, this study systematically investigated their combined effects under more realistic display configurations. The key findings are as follows: the RGB subpixel array exhibited the strongest gloss regardless of pixel–aperture ratio (RGB > PenTile ≈ RGBW), and the 50% aperture ratio was perceived as having stronger gloss than 100%. In particular, RGB50%, which exhibited the highest MTF in both vertical and horizontal directions, was perceived as having the strongest gloss, generally consistent with MTF superiority relationships. However, under conditions where the vertical and horizontal MTF superiority relationships did not match, some cases could not be explained solely by one-dimensional MTF characteristics, suggesting that spatial features arising from pixel structures may affect gloss perception in complex ways. Cluster analysis revealed individual differences in gloss perception, while image feature analysis suggested that pixel structure effects are more pronounced in images with higher frequency components. GLMM analysis supported the overall superiority of the RGB subpixel array observed in the average response rates, even when accounting for variability across observers and natural images as random effects.

The present findings have practical implications for display design. Notably, the GLMM analysis revealed that while a significant difference was observed between RGB100% and RGBW100% (*p* < 0.001), no significant difference was found between RGB100% and RGBW50% (*p* = 0.219). This suggests that adjusting the pixel–aperture ratio in displays with RGBW subpixel arrays may achieve gloss perception equivalent to that of RGB subpixel array displays. These results provide design guidelines for reducing visual quality differences among displays with different pixel structures, offering foundational knowledge for ensuring consistent shitsukan perception across increasingly diverse display technologies. Furthermore, our results indicate that gloss perception is broadly consistent with MTF superiority relationships, yet exceptions emerge when the vertical and horizontal MTF relationships disagree, suggesting the influence of spatial anisotropy. This implies that perceptual quality models for displays should incorporate two-dimensional spatial-frequency characteristics rather than relying solely on one-dimensional MTF. These findings may contribute to the development of perceptual metrics that more accurately predict shitsukan reproduction quality. From an application standpoint, they can support device-aware rendering or calibration strategies that adjust pixel–aperture ratio settings and image processing to reproduce consistent gloss (and shitsukan) across displays with different pixel structures. For example, in virtual reality environments where users wear head-mounted displays (HMDs) with varying display specifications, such strategies could help achieve more unified shitsukan perception across devices and improve the consistency of shared virtual experiences.

This study has several limitations. First, the experiment used only achromatic images, and the influence of color was not considered. Second, because self-emitting OLED displays generally have low sustained luminance, the 12 × 12 pixel simulation method used in this study cannot reproduce displays with very low aperture ratios, such as Micro-LED. Third, to reduce potential bias inherent in the 2AFC method, alternative designs that introduce an “indifference (neither)” option may be worth considering in future experimental designs.

Future work should include verification using higher-luminance self-emitting LED displays to address the limitation of the current simulation method. Additionally, analysis using more diverse image stimuli and a larger number of observers and investigation of the effects on other shitsukan attributes, such as roughness and transparency, are needed to further generalize the influence of display pixel structure on shitsukan perception. Verification using actual display panels with different pixel structures, as well as expanding the range of aperture ratio conditions and subpixel array types, also remains a challenge. Furthermore, two-dimensional MTF analysis to elucidate the effects of anisotropy suggested in this study is warranted.

## Figures and Tables

**Table 1:** List of stimulus pairs.

Pair ID	Stimulus Pair	
P01	RGB100%	RGB50%
P02	RGB100%	RGBW50%
P03	RGB100%	PenTile50%
P04	RGBW100%	RGB50%
P05	RGBW100%	RGBW50%
P06	RGBW100%	PenTile50%
P07	PenTile100%	RGB50%
P08	PenTile100%	RGBW50%
P09	PenTile100%	PenTile50%
P10	RGB50%	RGBW50%
P11	RGB50%	PenTile50%
P12	RGBW50%	PenTile50%

**Table 2:** Effect sizes between stimulus pairs for all responses. Asterisks indicate statistical significance (*: *p* < 0.05, ***: *p* < 0.001).

Stimulus Pair	P01	P02	P03	P04	P05	P06
*p*-Value	0.5688	0.0338 *	0.0149 *	0.0003 ***	0.0274 *	0.0854
Std. Dev.	11.35	12.68	14.18	11.85	11.87	15.11
Cohen’s *d*	0.13	0.50	0.58	0.89	0.52	0.40
Effect Size	Small	Medium	Medium	Large	Medium	Medium
**Stimulus Pair**	**P07**	**P08**	**P09**	**P10**	**P11**	**P12**
*p*-Value	0.0229 *	0.0904	0.6953	0.4656	0.0002 ***	0.8419
Std. Dev.	15.22	13.04	11.94	11.81	13.71	14.42
Cohen’s *d*	0.54	0.40	0.09	0.17	0.91	0.05
Effect Size	Medium	Medium	Very Small	Small	Large	Very Small

**Table 3:** Effect sizes for each cluster (only pairs showing significant *p*-values or effect sizes of “Very Large” or greater). Asterisks indicate statistical significance (*: *p* < 0.05, **: *p* < 0.01).

Cluster	GO1					
Stimulus Pair	P02	P03	P04	P07	P08	P11
*p*-value	0.057	0.095	0.089	0.052	0.078	0.001 **
Std. Dev.	9.97	19.05	12.43	17.68	15.92	13.19
Cohen’s *d*	1.38	1.18	1.21	1.41	1.26	3.13
Effect Size	Very Large	Very Large	Very Large	Very Large	Very Large	Huge
**Cluster**	**GO2**		**GO3**		**GO4**	
**Stimulus Pair**	**P04**	**P05**	**P04**	**P07**	**P04**	**P11**
*p*-value	0.0768	0.0735	0.1022	0.0026 **	0.0363 *	0.0019 **
Std. Dev.	12.86	11.71	11.95	10.54	11.65	11.87
Cohen’s *d*	1.26	1.28	1.15	2.61	0.64	0.98
Effect Size	Very Large	Very Large	Very Large	Huge	Medium	Large

**Table 4:** Image features for each natural image (normalized by maximum absolute value).

	GLCM Contrast	GLCM Energy	Hist Skewness	Hist Kurtosis	Mean Frequency	RMS Contrast
Plate	0.231	1.000	−1.000	−0.256	0.751	1.000
Silk	0.350	0.137	0.070	−0.398	0.904	0.551
Sink	0.488	0.269	0.603	−0.249	0.881	0.760
Gems	0.574	0.289	0.488	−0.787	0.743	0.904
Trumpet	1.000	0.386	0.238	−1.000	1.000	0.812

**Table 5:** Effect sizes for each natural image (only pairs showing significant *p*-values or effect sizes of “Very Large” or greater). Asterisks indicate statistical significance (*: *p* < 0.05, **: *p* < 0.01).

Natural Image	Plate		Silk			Sink
Stimulus Pair	P02	P11	P04	P05	P07	P04
*p*-value	0.0558	0.0421 *	0.0016 **	0.0125 *	0.0234 *	0.0204 *
Std. Dev.	12.38	10.83	9.11	13.53	13.32	9.87
Cohen’s *d*	1.08	1.15	2.01	1.48	1.31	1.35
Effect Size	Very Large	Very Large	Huge	Very Large	Very Large	Very Large
**Natural Image**	**Gems**		**Trumpet**			
**Stimulus Pair**	**P05**	**P06**	**P03**	**P04**	**P11**	**P12**
*p*-value	0.0281 *	0.0019 **	0.0058 **	0.0733	0.0049 **	0.0143 *
Std. Dev.	11.86	9.70	15.39	12.50	15.47	12.69
Cohen’s *d*	1.26	1.98	1.68	1.00	1.72	1.44
Effect Size	Very Large	Huge	Huge	Very Large	Huge	Very Large

**Table 6:** Fixed effect estimates from the GLMM analysis. Asterisks indicate statistical significance (***: *p* < 0.001).

Pixel Structure	β	Std. Err	*p*-Value
RGB100%	0.24	0.047	<0.001 ***
RGB50%	0.22	0.049	<0.001 ***
RGBW50%	0.11	0.059	0.067
PenTile100%	0.05	0.047	0.246
PenTile50%	0.05	0.071	0.487
RGBW100%	0 (Ref)	-	-

## Data Availability

The data presented in this study are available on request from the corresponding author due to restrictions.
